# Supplementary material for: Patterns of Intron Gain and Loss in Fungi
Source: PLoS Biol. 2004 Nov 30;2(12):e422. doi: 10.1371/journal.pbio.0020422 (PMC532390; doi:10.1371/journal.pbio.0020422)
Supplement: Table S1 — Also available at http://genes.mit.edu/NielsenEtAl/. (4.3 MB ZIP). [file pbio.0020422.st001.zip › NielsenEtAl/html/1275.html]

AN0910.1.NCU03695.1.MG09213.1.FG01865.1


```
 CLUSTAL W (1.82) Multiple Sequence Alignments - Introns Inserted


Sequence 1: NCU03695.1	532 aa
Sequence 2: FG01865.1	535 aa
Sequence 3: MG09213.1	536 aa
Sequence 4: AN0910.1	547 aa
Alignment Length: 562 aa
Number Identitical Residues: 242 aa
Alignment Score (without introns) 11178


MG09213.1 	MASTI-AARTRLVARSRPMHTYS-STYLYSRAPPPSRACFPSGGAGSGAGGSRCYSTHET
NCU03695.1	------MASFQLGPVSARVRVSTLSSTAFGRCPTSTRFTRLEIPFSASTRAPPAH--QMF
FG01865.1 	MAPTLGHGSSQLGSHMLRSSFYTHGRYATCGSPSASRHMLQALTKSRCLARPSASSIRTF
AN0910.1  	MASGLFSYSAPLRRNLACLSKERIALSDMVSFGPPERLRAGVRFFSKSRPSYNNNQKQNS
          	 :.        *            .        .. *        .         . :  

MG09213.1 	P----KRPKFTSRLRSAWKNSKITWYNIPVGVGIGFLGLVQFYRVQRR---EKDRLKDEA
NCU03695.1	G-----RRFFSNHMPKRPR---MQWHPIPIGLGIGLLGVMQFYKVYTRDRDEKRQAAVE-
FG01865.1 	ASNSGKRPRFSQRLGEAMRNSKIQWYQIPVGLGVGFLGLVQFYKVSSR---EKERIEME-
AN0910.1  	S----TKSSFGSRLRFALRNTKVEWYPIPVGLGIGLLGILHYYKSQRNER-IRQEREAEA
          	     .:  * .::    :.:.: *: **:*:*:*:**::::*:   ..   : .   *:

MG09213.1 	DADADSLSRPKKRQRIRPDGPW2QVQIMSTLPLKALSRLWGRFNELTLPYYLR~IPGFKL
NCU03695.1	GSEPGQAGKPKKRRRIRPDGPW2QVQVMSTLPLKAMSRLWGKFNELTIPYYLR~APGFRL
FG01865.1 	DGQEG-TRTPKKRARIRPDGPW2QVQVMSTLPLKAISRLWGRFNELTIPYYLR0---WQI
AN0910.1  	AGESFDFSKPPPRPKIRPSGPW~QVQIMSTLPLKAMSRLWGRFNEIELPYYLR~VPGFKL
          	 .:  .   *  * :***.*** ***:********:*****:***: :*****  ..:::

MG09213.1 	YSFIFGVN2LDEIEEQDLHNFPNLASFFYRTLKPGARVLDPNPLALLSPSDGRVLQYGQI
NCU03695.1	YAYFFGVN2LDEVEEQDLHKFPNLAAFFYRTLKPGVRPVDPNHNALVSPSDGRILQFGRI
FG01865.1 	HAN--ELS~LDEVAEPDLHVYPNLASFFYRTLKPGARPLDQDPHTLVCPSDGKVLQYGQI
AN0910.1  	YSWIFGVN~LDEVAEPDLHVYPNLAAFFYRKLKPGVRPLDPDPHAILSPSDGRILQFGLI
          	::    :. ***: * *** :****:****.****.* :* :  :::.****::**:* *

MG09213.1 	EGGDIEQVKGMTYSIDALLGETTP--SRPGSLASSGTSTPNPAQ-----DDEIVKQDEEF
NCU03695.1	EGGDIEQVKGMTYTIDALLGQNSPTPSISGTTLLDESTKAKSVNGDFEGDEELVKKDEEF
FG01865.1 	QGGDIEQVKGMTYSIDALLGKNTPPASVSGTSGTSTPATSLASN-DVSDEEALVKQDAEF
AN0910.1  	ERGEVEQVKGVTYSLDALLGSATPSQADHSKKFMDHHNEPSQKD------AASIAADEQF
          	: *::*****:**::*****. :*. :  ..   .    .   :         :  * :*

MG09213.1 	ARVNGISYTLPNLLSGPSSKSNKTDQDLE--DQSVTPSPASVSQVRADLELGE-KPWWSL
NCU03695.1	ARVNGISYTLPDLLSG----SKKHDKLFKPKDESVTPSKTSETEVSAELALGE-KPWYDV
FG01865.1 	AQVNGISYTLPDLLSG----TGKRGPSVK--DESMPASPGTVSEVSAELALGE-RPWYDV
AN0910.1  	ATMNGISYTLPSLFSG---ETGGLRKRSSSKDASTESQITSETLVREDLARGDGTPWYAP
          	* :********.*:**   .:       ...* *  ..  : : *  :*  *:. **:  

MG09213.1 	LAEQRRTKLYYAVIYLAPGDYHRFHSPTNWVVERRRHFAGELYSVSPYLQRTLPGLFTLN
NCU03695.1	LSGGERHTLYYAVIYLAPGDYHRFHSPTEWVVERRRHFAGELFSVSPYLQRTLPGLFTLN
FG01865.1 	VSPDKTTALYYAVIYLAPGDYHRFHSPTNWVVDRRRHFAGELYSVSPYLQRTLPGLFTLN
AN0910.1  	KPTSN-NALYYVVIYLAPGDYHRFHSPVPWVVESRRHFAGELFSVSPYLQRHLPGLFTLN
          	 .  .   ***.***************. ***: ********:******** ********

MG09213.1 	ERVVLLGRWRYGFFSYVPVGATNVGSIKINFDRELRTNSLTTDTAADRAAEEAARRGEPY
NCU03695.1	ERVVLLGRWRWGFFGYVPVGATNVGSIKINFDRELRTNSLTTDTAADRAAELAAAKGEPY
FG01865.1 	ERVVLLGRWRWGFFSYVPVGATNVGSIVVNFDKELRTNSLLTDTAADRAAEEAANRGEVY
AN0910.1  	ERVVLLGRWRWGFFSYTPVGATNVGSIKINFDSELRTNSLLTDTAADIAAAEAARRGEQY
          	**********:***.*.********** :*** ******* ****** **  ** :** *

MG09213.1 	SGYAEATYENASRVLHGHALRRGEEMGGFQLGSTVVLVFEAPAPR---MEGG----QVQD
NCU03695.1	LGYAEATYEAASPVLKGHALRRGEEMGGFQLGSTIVLVFEAPVAE---HDESG---KVTK
FG01865.1 	QGFAEATYEAASPILRGHALRRGEEMGGFQLGSTIVLVFEAPANK---VGEN----NQHI
AN0910.1  	PGFAEATYLHASRTLGGHPLQRGEEMGGFQLGSSIVLVFEAPMGTRKSFDAGWKEGQRDG
          	 *:*****  **  * **.*:************::*******    .:   . ...:   

MG09213.1 	GFVWAVEKGQTVKMGQALGRVVDDL
NCU03695.1	GWTWDVEKGRKIKMGQTLGHVAL--
FG01865.1 	GWDWAVEKGQQVKMGQALGRVVE--
AN0910.1  	GLNWTIEKGQRIKMGQKVAYVDIKE
          	*  * :***: :**** :. *  .
```
